# Cultural competence of nursing educators at medical universities of 2nd regional planning in Iran

**DOI:** 10.1186/s12909-023-04274-5

**Published:** 2023-05-11

**Authors:** Sahand Majnoon, Vivian M. Yates, Hossein Asgarpour, Ahmad Mirza Aghazadeh Attari, Mojgan Lotfi

**Affiliations:** 1 Department of Nursing, Tabriz University of Medical Sciences, Tabriz, Iran; 2 Department of Nursing, Cuyahoga Community College, Cleveland, OH USA; 3 Department of Nursing, Çanakkale Onsekiz Mart University, Çanakkale, Turkey; 4 Department of Basic Sciences, Paramedical Faculty, Tabriz University of Medical Sciences, Tabriz, Iran; 5 Medical Education Research Center, Tabriz University of Medical Sciences, Tabriz, Iran

**Keywords:** Cultural competence, Transcultural nursing, Nursing educators

## Abstract

**Background:**

One of the facets of nursing care, as a holistic profession, is cultural care. Considering the role of culture in individuals’ health behaviors, nurses are recommended to be mindful of cultural care. Since nursing educators should be culturally competent to teach cultural care to students, this study aimed to determine the cultural competence of nursing educators of medical sciences universities in the 2nd regional planning in Iran.

**Methods:**

The current research was a descriptive and survey study framed within Campinha-Bacote’s cultural competency model. All nursing educators of universities of medical sciences in the 2nd regional planning of Iran (Tabriz, Urmia, Ardabil, Khoy, Maragheh, Sarab, and Khalkhal) were considered as research units, and the cultural diversity questionnaire for nursing faculties (CDQNE-R) was sent to them. Out of 129 questionnaires sent, 84 were turned back. The data were analyzed by the SPSS 22 software.

**Results:**

The results of this study showed that the research participants agreed with the subscales of cultural awareness, cultural knowledge, cultural skill, and cultural desire according to Sealey and Yates’ interpretation scale. Also, the research units cast doubts on the cultural encounter subscale. The mean scores of the participants’ responses to the questions of every subscale equaled 4.11, 3.52, 3.71, 3.38, and 3.93 for the subscales of cultural awareness, cultural knowledge, cultural skill, cultural encounter, and cultural desire, respectively. Likewise, the mean scores of participants’ responses to the subscales of transcultural educational behaviors and general cultural competence equaled 3.90 and 3.73.

**Conclusions:**

The nursing faculties participating in the present study agreed with the 4 sub-models of Campinha-Bacote cultural care and the presence of cultural competence criteria. Also, the research units had doubts about the cultural encounter subscale. This result means that the research participants were undecided about their level of participation in face-to-face interactions with people from different cultural, racial, and ethnic groups. According to the results of the study, it is important to hold transcultural nursing training workshops and courses to maintain and improve the level of cultural competence of nursing faculties at universities of medical sciences in the 2nd regional planning in Iran.

## Background

Nursing is defined as a scientific and human profession and a discipline that focuses on the human care phenomenon and activities, helping individuals and groups significantly protect their health in cultural terms and get along with disability and death [1, 2]. Care refers to actions that directly help individuals and groups with overt and predicted needs to improve their living conditions or death encounters. In this respect, it is imperative to accentuate patients’ cultures to offer desirable care [2]. Culture refers to values, beliefs, customs, and symbols that shape individuals’ lifestyles and are transferred through interpersonal interactions. In other words, culture incorporates the values, beliefs, and learned and transferred life skills of a certain group and directs its thoughts, decisions, and performances within a distinct pattern [2, 3].

Nurses encounter patients with various cultures every day. Identifying these cultures in a region helps nurses offer effective therapeutic care. Salimbene points out that many health care providers who in the past served people of their own race are now expected to provide care to people of different races and languages ​​who have different health beliefs. Many studies have foregrounded considering patients’ cultures as an undeniable aspect in examining care needs and planning for culture-based nursing care [1, 4–7]. For example, Leininger, a specialized nurse in the intercultural nursing domain, argues that cultural beliefs, values, norms, and care models have enormous effects on disease survival, development, and circumstances and humans’ health and well-being feelings. Also, she mentions that if professional nursing care is not aligned and adjusted with care recipients’ beliefs and values, it leads to cultural conflicts, maladjusted behaviors, cultural stress, and imposition [7].

Nurses’ success in their professional performances and occupational circumstances depends on their capacity to conform to these multicultural conditions and further attention to the cultural category. This capacity in nurses is called cultural competence, which is defined by some nursing researchers as a process where the nurse continuously endeavors to acquire the ability to work with individuals, families, and communities in a context with various cultures [3].

Nurses’ acquisition of cultural competence calls for properly teaching the stages of cultural competence and facilitative and debilitative factors in cultural competence acquisition. University years play a significant role in developing attitudes and acquiring the knowledge and skills of cultural care. Some respective studies that have tackled the caring capacities of B.S. nursing students have reported their low cultural competence [1, 8, 9]. Thus, nursing educators should teach students the stages of cultural competence acquisition and correct approaches to cultural care during educational courses. Leininger introduces the discussion on nursing faculties’ competence in teaching intercultural nursing as the most sensitive problem in the agreement of nursing curriculum with the intricacies of the diverse modern society [1, 10].

Numerous studies have underscored teaching the stages of cultural competence to nurses in educational courses [9, 11, 12]. Likewise, there is a bulk of research attempting to promote the cultural competence of nursing educators. For instance, Ryan Twibell, Miller, and Brigham reported a project where nursing faculties sought to enhance their capacities in teaching intercultural nursing [1, 13]. However, a few studies have tested the knowledge and preparedness of nursing faculties in teaching intercultural nursing [14]. The chairmen and managers of nursing curricula in Florida in the United States conducted a study to improve and modify cultural diversity in nursing education programs. They found that, according to respondents’ mindsets, the lack of cultural knowledge, sensitivity, and awareness were among the most paramount problems they faced [15]. Hence, in planning to promote cultural care in culturally diverse societies, it is extensively crucial to examine the adequacy of nursing educators’ knowledge, sensitivity, and awareness in this area [11].

**Fig. 1:**
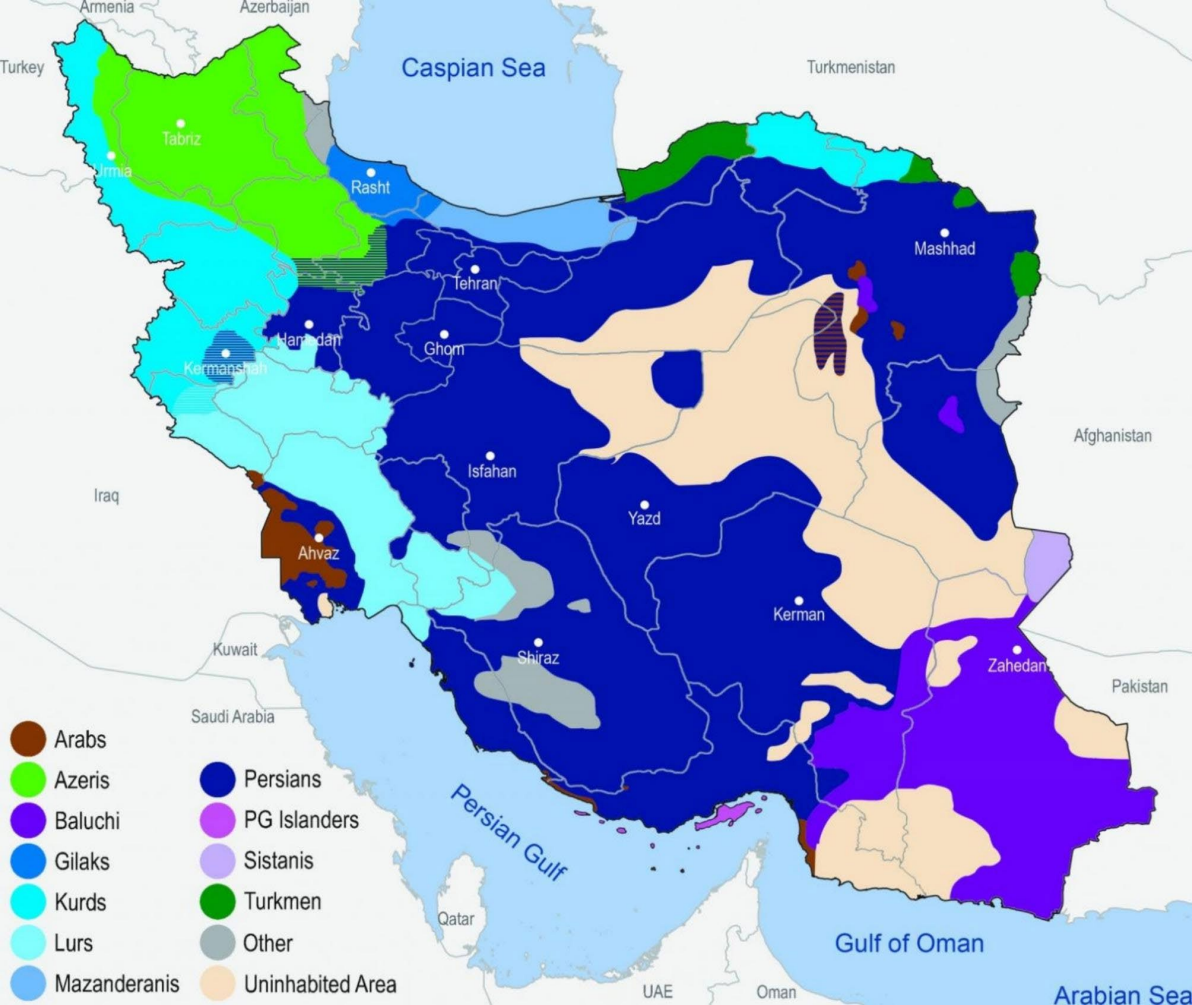
Map of Iran according to ethnic and cultural diversity of people in its different geographies

Iran is a country with cultural, ethnic, and racial diversity observed in its different locales. The presence of several Turk, Fars, Baluch, and Lor nations and their specific languages are manifestations of this cultural diversity, which is intensified by migrations from villages to cities and from towns to metropolises, especially in some regional planning zones (Fig. 1) [16].

Regional planning is determined Iranian zones used for land use purposes to examine resources and plan to develop the country [17]. In this regard, the tenfold regions of the Iranian universities have been determined and categorized for the country’s educational planning. According to this categorization, the second regional planning in Iran encompasses the Universities of Tabriz, Urmia, Ardabil, Khoy, Maragheh, Sarab, and Khalkhal. Cultural diversity is high in this region due to the presence of various ethnic and racial groups and its proximity to the boundaries of Iraq, Turkey, Azerbaijan, and Armenia. Therefore, considering past studies and the significance of cultural competence for nurses and multicultural conditions in the country, it is indispensable to measure nursing educators’ and faculties’ degrees of cultural competence.

Despite the significance of the topic and the research on nursing educators’ cultural competence in other parts of the world, no study has examined nursing educators’ degrees of preparedness for teaching intercultural nursing. Therefore, the present study embarked on determining the cultural competence of nursing educators at medical sciences universities in the 2nd regional planning in Iran.

## Methods

The present descriptive and survey study, conducted in the 2020–2021 period, aimed to determine the cultural competence of nursing educators at medical sciences universities in the second regional planning in Iran (Universities of Tabriz, Urmia, Ardabil, Khoy, Maragheh, Sarab, and Khalkhal). It was implemented after the acquisition of the ethics code from the Regional Committee of Ethics in Research at Tabriz University of Medical Sciences (IR.TBZMED.REC.1399.739).

The census method was used for sampling. All nursing educators and faculty members (A total of 129 instructors) teaching different nursing courses in faculties, laboratories, or hospital wards were selected. The inclusion criteria were teaching nursing at the considered universities and having teaching experience of above 2 years in the nursing field. The exclusion criterion was being only engaged with managerial responsibilities in the past two years. In this research, the researcher had no control over the environment, and the study was implemented in a completely normal milieu. After communicating the research procedure to the examined units and acquiring their informed consent, the researcher sent them the questionnaires through emails or virtual apps and did not intrude on the natural environment. Likewise, trusteeship in using resources and the confidentiality of the participants’ information were thoroughly observed.

## Data collection instruments and procedure

The Cultural Diversity Questionnaire for Nursing Educator-Revised (CDQNE-R) was used to collect data. CDQNE-R is a dichotomous instrument designed by Sealy based on Campinha-Bacote’s cultural competence model [1]. With the permission of the instrument designer, Vivian Yates modified and improved CDQNE. The permission to use this instrument in this research was acquired from Vivian Yates through an email. As mentioned, CDQNE-R has two sections. The first section, with 9 items, elicits demographics and nursing specializations, and the second section, with 41 items, determines nursing faculties’ degree of cultural competence through a five-point Likert scale ranging from strongly agree (score 5) to strongly disagree (score 1). Notably, the participants’ responses were scored based on a five-point Likert scale involving strongly disagree (1), disagree (2), undecided (3), agree (4), and strongly agree (5). In this questionnaire, scores 1 and 5 indicate the lowest and highest desirability. The questionnaire designer used the scale below to interpret the responses: <1.5 = strongly disagree (SD), 1.5–2.5 = disagree (D), 2.5–3.5 = undecided (U), 3.5–4.5 = agree (A), and > 4.5 = strongly agree (SA). The second section includes five subscales, including cultural awareness, cultural knowledge, cultural skill, cultural encounter, and cultural desire. Cultural awareness means self-examination and accurate and deep knowledge of a person’s cultural and professional background. Cultural knowledge is the process by which people seek to find the content of different cultures. Cultural skill is the ability to gather cultural data relevant to the patient’s current problem as well as perform an accurate culture-based physical assessment. Cultural encounter is a process that encourages healthcare providers to directly engage in cross-cultural interactions with patients from culturally diverse backgrounds. Cultural desire is the motivation of the healthcare provider to want to be involved in the process of cultural awareness, cultural knowledge, cultural skill and familiarization with cultural exposure, rather than being forced [18, 19]. The subscale of intercultural educational behaviors measures the degree of inclusion of transcultural nursing concepts in the educational content of the nursing curriculum by nursing educators [20]. Furthermore, the intercultural educational behaviors subscale involves 11 items distributed among other subscales [20].

Although the instrument has been entitled by the term *cultural diversity*, all studies using the CDQNE-R and CDQNE questionnaires, including Sealey’s quest, i.e., its designer, have applied it for measuring cultural competence, and the term *diversity* is just employed for its title.

At first, the list of nursing faculties whose information fit the inclusion criteria was received from the considered universities. Then, an email, including the CDQNE-R questionnaire, an informed consent form, and a cover letter, was sent to all participants (129 nursing educators employed in the considered universities) via a link from the Porsline site. One month later, a reminder was sent to those who had not filled out the questionnaires. The second reminder was sent to the research units that had not responded to the questionnaires one month after the first reminder.

Yates confirmed the validity of this questionnaire in a study entitled *cultural competence levels of Ohio associate degree nursing educator* [20]. After the instrument was translated into Persian in this research, two English language specialists were asked to confirm the agreement of the rendered scale with the original one. Furthermore, five nursing faculties at Tabriz University of Medical Sciences, who were active in the cultural competence domain, helped confirm the instrument’s content validity. After the collection of suggestions and the necessary modifications, the final instrument was adjusted and employed. Yates also measured the reliability of CDQNE-R and estimated its Cronbach alpha coefficient at 0.93[20]. It is worth noting that this coefficient was obtained at 0.92 in the present study.

### Data analysis

Descriptive statistics (frequency, percentage, mean, and standard deviation) and inferential statistics (Pearson correlation coefficient and multiple linear regression) were used to examine the data in line with the purposes of the study. The data were analyzed by the SPSS 22 software, and the maximum significance level was considered at 5% if necessary.

## Results

The collected data were analyzed by descriptive and inferential statistical methods. Table 1 presents the demographics of the research units, such as gender, age, ethnical-racial backgrounds, occupational states, scientific ranks, university or faculty they teach, teaching level, teaching experience, highest degree received, and specialized subfield.

**Table 1:** Demographics of the participants

Characteristic		No.	Frequency (%)
Age	< 30	9	10.7
	31–40	22	26.2
	41–50	34	40.5
	51–60	19	22.6
	> 61	0	0
Gender	Female	55	65.5
	Male	29	34.5
Cultural-ethnic background	Fars	6	7.1
	Turkish	75	89.3
	Kurd	3	3.6
Occupation state	Full-time	38	45.2
	Service period and commitment	17	20.2
	Contractual	22	26.2
	Temporary/under-a-contract	7	8.3
Academic rank	Full professor	4	4.8
	Associate professor	15	17.9
	Assistant professor	26	31
	Instructor	39	46.4
Service university	Tabriz	36	42.9
	Urmia	18	21.4
	Ardabil	19	22.6
	Maragheh	4	4.8
	Khoy	5	6.0
	Sarab	0	0
	Khalkhal	2	2.4
Teaching level	B.S.^1^	33	39.3
	M.S.^2^	6	7.1
	Ph.D.^3^	1	1.2
	B.S. and M.S.	29	34.5
	M.S. and Ph.D.	1	1.2
	B.S., M.S., and Ph.D.	14	16.7
Teaching experience	≥ 5	12	14.3
	6–10	22	26.2
	11–15	20	23.8
	16–20	19	22.6
	21–25	2	2.4
	26–30	9	10.7
	≥ 31	0	0
Highest degree	M.S.	37	44.0
	Ph.D.	47	56.0
Subfield	Medical-surgical nursing	52	61.9
	Community health nursing	9	10.7
	Pediatric nursing	10	11.9
	Maternal newborn nursing	2	2.4
	Psychiatric nursing	7	8.3
	Women’s health	0	0
	Nursing management	1	1.2
	Transcultural nursing	0	0
	Gerontology	1	1.2
	Emergency nursing	2	2.4

In line with the specific purpose of the study, i.e., determining the (transcultural) intercultural educational behavior, knowledge, awareness, skill, encounter, and desire of nursing educators at medical sciences universities of the 2nd regional planning in Iran, the means and standard deviations of the participants’ responses to every subscale item of CDQNE-R were estimated and presented in Table 2. The questionnaire designer used the scale below to interpret the responses: <1.5 = strongly disagree (SD), 1.5–2.5 = disagree (D), 2.5–3.5 = undecided (U), 3.5–4.5 = agree (A), and > 4.5 = strongly agree (SA).

**Table 2:** Evaluating subscales among nursing educators of medical sciences universities in 2nd regional planning of Iran

Item		Mean *	SD	Response categorization ∗∗
Cultural awareness	7. I am aware that biological variations exist in different cultural, racial, and ethnic groups.	3.75	1.05	A
	10. When I care for a client, I consider how the difference between our perceptions of health, illness, and preventive health could affect the outcome of my care.	4.36	0.70	A
	28. I teach my students that the client’s culture is a determining factor in the client’s perception of health and illness and in his or her adherence to the prescribed treatment regimen.	4.43	0.66	A
	31. I encourage my students to examine their attitudes, preconceived notions and feelings toward members of other cultural/racial/ethnic groups.	4.00	0.76	A
	36. I teach my students that when working with clients who are culturally, racially, or ethnically different they should become familiar with indigenous beliefs and practices.	4.08	0.85	A
	37. I believe that failure to explore my own culture’s influence on the way I think and behave may lead me to impose my own values and beliefs on my clients.	4.07	0.91	A
	38. What I believe about health, illness, and preventative care is influenced by my culture.	4.08	0.82	A
	40. I accept that male-female roles may vary among significantly among different cultures and ethnic groups.	4.17	1.14	A
Cultural knowledge	5. I am knowledgeable about variations in drug metabolism among specific cultural groups.	2.90	1.01	U
	11. I am knowledgeable about the biological variations that exist among specific cultural, racial, and ethnic groups.	3.56	1.13	A
	14. I am knowledgeable about diseases that have a high incidence among cultural/racial/ethnic groups in our service area.	3.44	0.99	U
	16. I require that students be knowledgeable about diseases that have a high incidence among clients in our service area from diverse cultural, racial, and ethnic groups.	4.10	1.02	A
	17. I have a clear understanding of the differences in meaning of the following terms; acculturation, assimilation, and socialization.	3.39	1.08	U
	21. My students are expected to demonstrate knowledge of their client’s world views, beliefs, and practices by incorporating this knowledge in their plans of care.	3.62	1.06	A
	22. I am knowledgeable about diseases that are common in the countries of origin of recent immigrants in our service area.	3.05	1.06	U
	29. I am knowledgeable about the socio-economic and environmental risk factors that contribute to the major health problems of culturally, ethnically, and racially diverse populations served by my nursing program.	3.70	0.90	A
	32. I know the prevailing beliefs, customs, norms, and values of the cultural/racial/ethnic groups, other than my own, residing in our service area.	3.81	0.78	A
	35. I am knowledgeable about the population percentages of the major ethnic groups living in my service area.	3.23	1.05	U
	39. I have a clear understanding of the differences in meaning of the following terms; immigrant, alien resident, and citizen.	3.94	1.01	A
Cultural skill	1. I feel confident in using a variety of cultural assessment tools in the health care setting.	3.32	1.27	U
	8. I use the appropriate communication style and protocol to communicate with clients who are of different cultural/racial/ethnic backgrounds.	4.08	0.90	A
	9. My students are required to seek information on acceptable behaviors, courtesies, customs, and expectations that are unique to the culturally, racially, and ethnically diverse groups served by our program.	3.80	1.01	A
	12. I am knowledgeable of keywords and phrases needed to communicate effectively with the major groups with limited English language proficiency that are served by our program.	3.58	0.86	A
	18. I am confident that I possess the necessary skills and experience to select and work with appropriate translators as needed to care for clients with limited English language proficiency	3.55	0.99	A
	33. I teach my students to recognize presenting signs and symptoms as they are manifested in individuals who are culturally, racially, and ethnically diverse.	3.98	0.80	A
	34. The cultural assessment tool that I use elicits information about clients’ dietary practices, health beliefs, and social organization.	3.33	1.09	U
Cultural encounter	3. I am involved socially with cultural/racial/ethnic groups different from my own, outside of my teaching role and health care setting.	4.00	1.03	A
	13. I seek out clinical opportunities for my students to care for clients who are culturally, racially, and ethnically diverse.	3.70	0.94	A
	15. I am in contact with individuals who provide health services to groups that are culturally, racially, and ethnically diverse.	3.15	1.21	U
	20. I attend holiday celebrations within culturally, racially and ethnically diverse communities.	2.83	1.14	U
	23. I have spent extended periods of time (i.e. at least seven consecutive days) living among people from cultural/racial/ethnic groups different from my own.	2.77	1.29	U
Cultural desire	30. I patronize businesses on my service area that are owned by people who are culturally, racially, and ethnically diverse.	3.87	0.86	A
	2. I make time to include cultural competence in my course content.	3.74	0.98	A
	4. Caring for clients who are culturally, racially, or ethnically diverse is a challenge that I welcome.	4.20	0.86	A
	6. I avail myself of professional development and training opportunities to enhance my knowledge and skills in the provision of health care services to culturally, racially, and ethnically diverse groups.	3.83	1.01	A
	19. My students are required to seek information on acceptable behaviors, courtesies, customs, and expectations that are unique to the culturally, racially, and ethnically diverse groups served by our program.	3.42	88.0	U
	24. I screen books, movies, and other media sources for negative cultural, racial, or ethnic stereotypes before using them in my course or sharing them with clients cared for by me or by my students.	3.45	1.29	U
	25. I am personally and professionally committed to providing nursing care that is culturally competent.	4.29	0.88	A
	26. I am personally and professionally committed to teaching how to provide nursing care that is culturally competent.	4.19	0.76	A
	27. I advocate for the review of my program’s mission statement, goals, policies and procedures to ensure that they incorporate principles and practices that promote cultural and linguistic competence.	4.36	0.75	A
(Transcultural) intercultural educational behaviors	2. I make time to include cultural competence in my course content.	3.74	0.98	A
	9. My students are required to seek information on acceptable behaviors, courtesies, customs, and expectations that are unique to the culturally, racially, and ethnically diverse groups served by our program.	3.80	1.01	A
	13. I seek out clinical opportunities for my students to care for clients who are culturally, racially, and ethnically diverse.	3.70	0.94	A
	16. I require that students be knowledgeable about diseases that have a high incidence among clients in our service area from diverse cultural, racial, and ethnic groups.	4.10	1.02	A
	21. My students are expected to demonstrate knowledge of their client’s world views, beliefs, and practices by incorporating this knowledge in their plans of care.	3.62	1.06	A
	24. I screen books, movies, and other media sources for negative cultural, racial, or ethnic stereotypes before using them in my course or sharing them with clients cared for by me or by my students.	3.45	1.29	U
	26. I am personally and professionally committed to teaching how to provide nursing care that is culturally competent.	4.19	0.76	A
	28. I teach my students that the client’s culture is a determining factor in the client’s perception of health and illness and in his or her adherence to the prescribed treatment regimen.	4.43	0.66	A
	31. I encourage my students to examine their attitudes, preconceived notions and feelings toward members of other cultural/racial/ethnic groups.	4.00	0.76	A
	33. I teach my students to recognize presenting signs and symptoms as they are manifested in individuals who are culturally, racially, and ethnically diverse.	3.98	0.80	A

*Every item mean has been estimated as strongly agree (5), agree (4), undecided (3). Disagree (2), and strongly disagree (1), according to the scoring scale of the questionnaire designer

** Every response was categorized as strongly agree (SA), agree (A), undecided (U). Disagree (D), and strongly disagree (SD), based on the interpretive categorization of the questionnaire designer

The results associated with the cultural awareness subscale showed that the highest score belonged to Item 28 (I teach my students that the client’s culture is a determining factor in the client’s perception of health and illness and in his or her adherence to the prescribed treatment regimen.), with a mean of 4.43. Item 7 (I am aware that biological variations exist in different cultural, racial, and ethnic groups), with a mean score of 3.75, allocated the lowest score to itself. All items of this subscale fell into the cultural agreement category. The mean score of the cultural awareness subscale was estimated at 4.11 ± 0.52, indicating the high cultural awareness of the participants.

The results related to cultural knowledge showed that the highest score belonged to Item 16 (I require that students be knowledgeable about diseases that have a high incidence among clients in our service area from diverse cultural, racial, and ethnic groups), with a mean score of 4.10. The lowest score was assigned to Item 5 (I am knowledgeable about variations in drug metabolism among specific cultural groups), with a mean of 2.90. The respondents revealed their agreement with six items and hesitated on the other five items of this subscale. The mean score of the cultural knowledge subscale was estimated at 3.52 ± 0.61. Thus, this subscale with a trivial difference fell into the cultural agreement domain.

Concerning the cultural skill subscale, the results displayed that the highest score was related to Item 8 (I use the appropriate communication style and protocol to communicate with clients who are of different cultural/racial/ethnic backgrounds) with a mean of 4.08, and the lowest score belonged to Item 1 (I feel confident in using a variety of cultural assessment tools in the health care setting) with a mean of 3.32. The mean score of the cultural skill subscale was computed at 3.71 ± 60, and this subscale fell into the cultural agreement category.

The results on the cultural encounter subscale revealed that many participants in the research agreed with Item 3 (I am involved socially with cultural/racial/ethnic groups different from my own, outside of my teaching role and health care setting). The mean score of this item equaled 4. Likewise, many hesitated about Item 23 (I have spent extended periods of time (i.e. at least seven consecutive days) living among people from cultural/racial/ethnic groups different from my own), with a mean score of 2.77. The total mean score of this subscale equaled 3.38 ± 0.71. Hence, it is observed that many of the examined research units cast doubts on encounters with various cultural groups.

The outcomes related to cultural desire displayed that the highest score of this subscale belonged to Item 27 (I advocate for the review of my program’s mission statement, goals, policies and procedures to ensure that they incorporate principles and practices that promote cultural and linguistic competence), with a mean score of 4.36. Item 19 (I keep abreast of the major health concerns and issues of culturally, racially, and ethnically diverse client populations residing in my program’s service area), with a mean score of 3.42, allocated the lowest score to itself. That is to say, 2 items out of 8 were at the cultural doubt level, and the rest fell into the cultural agreement category. The mean score of this subscale, on which many participants agreed, was estimated at 3.93 ± 0.54.

The results derived from examining intercultural educational behaviors showed that the highest score was associated with Item 28 (I teach my students that the client’s culture is a determining factor in the client’s perception of health and illness and in his or her adherence to the prescribed treatment regimen), with a 4.43 mean. The lowest score belonged to Item 24 (I screen books, movies, and other media sources for negative cultural, racial, or ethnic stereotypes before using them in my course or sharing them with clients cared for by me or by my students) with a mean score of 3.45. Only did one item fall into the doubt category, and others remained at the agreement level. The total mean of the intercultural educational behaviors items was calculated at 3.90 ± 0.55.

According to Campinha-Bacote’s cultural competency model, used as a framework in this research, the total value of cultural competence fell into the cultural agreement category based on the estimated means of cultural awareness (3.71), cultural desire (3.93), cultural skill (3.71), cultural knowledge (3.52), and cultural encounter (3.38). This implies that the participants agreed on their possession of awareness, skill, knowledge, and desire to offer culturally competent care and teach nursing students to provide cultural care. Besides, the participant nursing educators in this research cast doubt on their cultural interactions with different individuals. Tables 3 and 4 present the results of determining the relationship of the total cultural competence index with every subscale.

Table 3 displays the reciprocal relationship between the total cultural competence and the independent variables in the regression. With respect to the results of Table 4, if one unit is added to the scores of cultural awareness, cultural knowledge, cultural skill, and cultural encounter, the total cultural competence score will change by 25.6%, 22.1%, 24.3%, and 25.5%. The highest effect size belongs to the cultural awareness and encounter variables, and the cultural desire variable was omitted by the test.

**Table 3:** Correlation between cultural competence and its subscales

Subscale	Correlation coefficient (r)	Sig. (p-value)
Cultural awareness	0.68	< 0.001
Cultural knowledge	0.83	< 0.001
Cultural skills	0.82	< 0.001
Cultural encounter	0.86	< 0.001
Cultural desire	0.87	< 0.001

**Table 4:** Correlation between cultural competence and its subscales

Dependent variable	Regression coefficient (β)	Sig. (p-value)
Cultural awareness	0.256	< 0.001
Cultural knowledge	0.221	< 0.001
Cultural skills	0.243	< 0.001
Cultural encounter	0.255	< 0.001

## Discussion

Cultural competence has been defined as a process rather than an endpoint. In this process, nurses continuously endeavor to acquire information on the cultural content of individuals, families, and society in an effective manner [4, 18, 21]. The present study investigated the degree of cultural competence among nursing educators and faculties at medical sciences universities of the 2nd regional planning of Iran in 2021. Out of 129 nursing educators, 84 (65%) filled out the CDQNE-R questionnaire. The results on the demographics of the respondents showed that none of the participants had received transcultural nursing training. It seems that this subfield has been sensitively neglected at Iranian universities and nursing curricula. The transcultural nursing subfield focuses on examining different cultures with respect to health-disease values and beliefs to provide culture-based care [1].

The results of this study regarding the absence of transcultural nursing education are in line with the findings of the research by Sealey, who reported that only 3% of the participants had majored in transcultural nursing [1]. Leininger introduces the discussion on nursing faculties’ competence in teaching transcultural nursing as the most crucial problem in preparation for encountering challenges rooted in cultural diversity [10]. In a national survey in the United States, Kelly discovered that only 19% of nursing educators had received certifications to participate in a transcultural nursing course. She also suggested incorporating extra postgraduate programs in transcultural nursing in the curriculum to make new nursing educators ready for teaching this topic and playing role models to students [19].

The cultural awareness subscale embarks on recognizing the personal biases, prejudgments, and hypotheses of various individuals in cultural terms. The cultural awareness acquisition process is essential for the progress of cultural competence and is the most significant component in Campinha-Bacote’s cultural competency model [22]. In this research, the highest score belonged to the cultural awareness subscale (4.11), which was expectable since cultural awareness was constantly recognized as the most paramount element of cultural competence regarding past research [21, 23, 24]. In her study in 2003, Sealey aimed to determine the cultural competence of faculty of baccalaureate nursing programs in Louisiana in the United States and estimated the mean score of the cultural awareness subscale at 4.14. This subscale also allocated the highest score to itself in her study [1]. Yates [20] and Siswadi et al. [25] reported the mean score of cultural awareness at 4.36 and 4.28, which were the highest scores among other subscales. Unlike previous studies, the cultural awareness subscale was ranked third after the cultural knowledge and transcultural educational behaviors in Sandra Burns’ quest [26]. The mean score of the cultural awareness subscale was also ranked second after cultural desire in Chen’s study [27]. Using the CDQNE-R instrument, Baghdadi estimated the mean score of the cultural awareness subscale at 35.2, which was smaller than the cultural knowledge subscale [28, 29].

The cultural knowledge subscale measures the participant educators’ degree of knowledge of cultural competence concepts and is a process through which individuals look for the content of various cultures [22, 30]. The mean score of the respondents in the 11-item cultural knowledge subscale equaled 3.52 ± 0.6.

The review of the literature reveals that cultural knowledge extremely contributes to providing culturally competent care [22, 24, 31, 32]. Cultural knowledge is also significant in engaging nursing students in classes held on cultural diversity [20]. The cultural knowledge subscale equaled 3.75 in Yates’ study and, in line with Sealey’s study, fell into the cultural agreement category [20]. Siswadi et al. [25] reported the mean cultural knowledge score of the examined units at 3.81. In Burns’ study, the highest score belonged to the cultural knowledge subscale (44.60) [26, 28], while the lowest mean score was allocated to this subscale (2.62) in Chen’s study based on the IAPCC-R instrument [27]. Furthermore, cultural knowledge gained the highest score among other subscales in Baghdadi’s study [28, 29]. In the present study, cultural knowledge was ranked fourth among 5 subscales. This result can be due to the lack of sufficient attention to teaching concepts associated with the cultural competence process and transcultural nursing to nurses and nursing educators.

The cultural skills subscale refers to the ability to collect cultural data tied to the current problem of the patient and make a meticulous culture-based physical evaluation [22]. A healthcare provider should know that a patient’s physical, biological, and mental differences can influence the outcomes of the physical evaluation. These differences can manifest in body performance, skin color, observable physical characteristics, and laboratorial results [32].

The mean score of the 8-item cultural skill subscale equaled 3.71 ± 0.60. This result shows that the research units agree on possessing the necessary skills to culturally examine help-seekers and build relationships with nursing students and them. The cultural skill subscale mirrors educators’ convenience in using culture-evaluating tools, communicative styles in interacting with help-seekers with diverse cultures, and efficiency in evaluating culturally different help-seekers [33]. In their study, Jones et al. reported that when they asked nurses, who worked with Mexican help-seekers, what was their main problem as nurses, they referred to *establishing effective relationships* and *linguistic barriers* [34]. However, the highest score belonged to the cultural skill after the awareness subscale in this study. In her study, Sealey reported the mean score of the cultural skill subscale at 3.65, which was aligned with the results of the present research. This subscale fell into the agreement category in both studies. That is to say, the research units agreed on possessing skills for collecting information on the cultural background of help-seekers and students and evaluating them culturally. The findings of the study conducted by Yates [20] (mean = 3.79) correspond with the results of Sealey’s and our studies concerning the cultural skill subscale. Siswadi [25] reported the mean score of nursing educators’ cultural skills at Indonesian universities at 3.96, and the cultural skill subscale was ranked fourth in Chen’s study [27]. Marzilli reported the mean score of cultural skill at 3.54, which was the highest among the other subscales [24, 35].

In the present study, the second rank belonged to the cultural skill subscale, showing that the participant faculties were sufficiently prepared to evaluate help-seekers and students culturally. Due to the ever-increasing growth in the diversity of cultural and racial groups in different regions of Iran, it is imperative to attend to linguistic skills, such as the skill to work with different translators, in nursing educators and nurses.

The cultural encounter subscale reflects the interaction of the research units with different racial and cultural groups. The purpose of cultural encounters, as a prerequisite to cultural competence development, is to create a wide spectrum of responses to transcultural situations [36]. Direct interactions with patients of different cultural groups improve or alter individuals’ mindsets about a cultural group and can prevent likely prejudgments [22, 32]. The mean score of the research units in the 6-item cultural encounter subscale equaled 3.38 ± 0.71, indicating that the respondents were doubtful about their level of participation in face-to-face interactions with different cultural, ethnic, and racial groups. This subscale received the lowest score in this research among the other subscales and was the sole index falling into the doubt category. Lower scores may be attributed to the lack of opportunity or tendency to interact with individuals of other cultural groups. The cultural encounter also scored the lowest in Sealey’s study, similar to the present research [1]. In her research, Yates estimated the cultural encounter score at 3.34, which was at the doubt level [20]. Chen also reported the mean score of the cultural encounter subscale at 2.89. This value also fell into the doubt category according to Sealey’s interpretive scale [27]. In line with the results of the present research, the cultural encounter score was the lowest among the other subscales in many of the mentioned studies [25, 26, 29].

Ryan et al. believed that the lack of authentic experiences with diverse cultures is one of the barriers to developing a culture-centered curriculum. They also reported that the students engaged in a cultural interaction experience identified increased awareness of the need for cultural competence, familiarity with personal biases, and the need for perceiving various relational models as the main outcomes of this experience [13]. Considering the results of the study above and the significance of cultural encounters, as reflected by past studies, it is suggested that the chancellors of the Iranian medical sciences universities, nursing faculties, and the nursing managers and directors of the Ministry of Health increase the number of programs where nursing faculties can interact with different racial and cultural groups and pave the way for the participation of nursing faculties in these interactions.

The cultural desire subscale reflects healthcare providers’ motivation for voluntarily engaging in the process of cultural awareness, cultural knowledge, cultural skill, and cultural encounter. In the present research, the mean score of this subscale equaled 3.93 ± 0.54, indicating the participants’ cultural desire and commitment to provide care and teach racially and culturally different individuals. Sealey estimated the mean score of participants’ cultural desire at 3.67, which conformed to the findings of the present research [1]. The results of Yate’s study (mean = 4.10) were also in line with the findings of our and Sealey’s studies. This subscale, with a mean of 4.34, allocated the third rank to itself in Burns’ study [26], while the highest score belonged to the cultural desire subscale among the others in Chen’s study (mean = 3.57) [27].

Cultural desire is an important primary step in providing culturally competent care [20]. The lack of cultural desire can lead to nursing faculties’ non-commitment to accessing a transcultural cultural lesson plan and influence their perceptions, attitudes, and behaviors with individuals with various cultural backgrounds. This, in turn, can impact students’ shaped perceptions and attitudes as well [37, 38].

The transcultural educational behaviors subscale measures to what extent nursing educators have incorporated transcultural nursing concepts into the educational content of the nursing curriculum [20]. In this research, the mean score of the research units in this subscale equaled 3.90 ± 0.55, indicating that the participants agreed with inserting the transcultural nursing concepts into the educational content of their educational and clinical classes. Sealey, in her study, showed that the examined units, with a score of 3.97, agreed with incorporating transcultural nursing concepts into the educational content. Her findings, with a trivial difference, are in line with the results of our study [1]. Yates also reported aligning results with the findings of our and Sealey’s studies and estimated the mean score of participants at 4.06 in this subscale [20]. This result also reflects the agreement of the research units for including transcultural nursing concepts in the nursing curriculum [27].

The attainment of multicultural education calls for faculty members’ cultural competence and their efforts and commitment to planned educational strategies and opportunities that embrace diverse cultural concepts [20]. With respect to the results of the present study and past research, it is suggested that specialized educational courses be incorporated into the academic and clinical teaching content of nursing educators for the purpose of offering approaches that improve the instruction of transcultural nursing concepts.

The results on the relationship between the total cultural competence index and every one of its subscales among nursing educators showed that the maximum effect on the dependent variable was linked to the cultural awareness variable. In similar past studies, only Sealey employed multiple regression analysis to examine the effect size of dependent variables (cultural awareness, cultural knowledge, cultural skill, cultural encounter, and cultural desire) on the independent variable (total cultural competence), where the cultural knowledge, cultural skill, cultural encounter, and cultural desire explained 99% of the variance. Unlike her study, where the cultural awareness subscale was excluded from the model according to the analysis process (1), the cultural desire index was omitted from the model in our research.

This study estimated total cultural competence based on the mean scores of the cultural awareness, cultural knowledge, cultural skill, cultural encounter, and cultural desire indices in Campinha-Bacote’s cultural competency model [22]. According to this model, total cultural competence falls into the cultural agreement category. That is to say, the participants agreed that they maintained cultural awareness, skill, knowledge, and desire to provide culturally competent care and teach their nursing students how to provide cultural care. The ultimate results of studies by Sealey, Yates, and Siswadi, who also employed this model, were in line with the findings of the present research at the total cultural competence level [1, 20, 25]. In her study, Burns reported the total cultural competence score at 170.9. Considering Ume-Nwagbo’s interpretive scale, the examined units in this study were moderately competent in cultural terms [26, 28]. Likewise, Marzilli estimated the total cultural competence score at 162.3, and Texan nursing educators’ cultural competence was also moderate [35].

## Conclusions

The results of this research showed that the total cultural competence of the Iranian nursing educators and faculties in the 2nd regional planning with a high cultural diversity was in the agreement circumstance in a strongly agree-strongly disagree spectrum. Furthermore, cultural awareness and cultural desire extensively contributed to this outcome. Since providing culture-based care is necessary for reducing inequalities and enhancing justice in the country’s healthcare system, the cultural competence of nursing faculties is a key factor in the cultural training of nurses for preventing some consequences, such as racism, culture imposition, stereotyping, high likelihood of medical and nursing malpractice, and non-provision of thorough care. Nursing administrators of medical sciences universities should step toward recruiting culturally competent nursing educators to compensate for the lack of these forces to some extent. Likewise, concerning the general outcomes of the research, we suggest modifying the nursing curriculum to provide cultural competence and transcultural nursing training.

The results of this study are limited to the 2nd regional planning of Iran. Thus, more extensive studies on the nursing educators of state and Azad medical sciences universities in other planned regions of the country are recommended. Furthermore, future studies are proposed to examine transcultural nursing issues using qualitative and mixed methods.

## Data Availability

The datasets used and/or analyzed during the current study are available from the corresponding author on reasonable request.

## Footnotes

1 Bachelor of Science.

2 Master of Science.

3 Doctor of Philosophy.
